# Financial inclusion and farmers’ income increase: Effects and realization mechanism

**DOI:** 10.1371/journal.pone.0324815

**Published:** 2025-06-23

**Authors:** Guibo Liu, Xiaoxian Gong, Guandong Wang

**Affiliations:** 1 School of Economics, Shandong Women’s University, Jinan, Shandong, China; 2 School of Accountancy, Shandong Technology and Business University, Yantai, Shandong, China; 3 School of Economics, Shandong Women’s University, Jinan, Shandong, China; Sultan Qaboos University, OMAN

## Abstract

From the perspective of agricultural industrialization, based on China’s provincial data from 2006 to 2022, it studies the impact of financial inclusion on farmers’ income. We have found that financial inclusion can effectively promote the growth of farmers’ income, and this effect shows the heterogeneity of different income structures, income levels, and regional types. More importantly, agricultural industrialization is an important mechanism for financial inclusion to promote farmers’ income growth. However, the interactive effect of financial inclusion and agricultural industrialization has not promoted the growth of farmers’ income, indicating that there are certain structural problems in the current development of financial inclusion. We suggest that the development of financial inclusion should aim at the agricultural industrialization based on division of labor and cooperation. It can focus on supporting the development of local leading industries with regional comparative advantages, guide farmers to enter the agricultural industry chain, and improve farmers’ opportunities to share economic results.

## Introduction

From the perspective of welfare economics, the goal of agriculture and rural development is to improve the income level and quality of life of farmers. China has always attached great importance to rural economic development and regarded it as an important breakthrough to solve the problem of farmers’ low income. The per capita disposable income of farmers rose from RMB 9429.6 in 2013 to RMB 20133 in 2022, with an average annual growth rate of 8.79%. However, with the changes in the internal and external economic environment, the growth rate of farmers’ income has gradually slowed down, and the pressure on farmers’ income to continue to grow has gradually increased. This is manifested in the following aspects: first, China’s agriculture has been implementing extensive business model for a long time. Agricultural products are under the dual pressure of “price ceiling” and “cost floor” (Wang, 2016) [1]. The space for activities in the middle is getting smaller and smaller, and the income that farmers can obtain from agricultural production continues to decrease and the rising space is shrinking. Second, with China’s urbanization and industrialization gradually entering the middle and late stage, the continuous promotion and use of modern high and new technologies, and the impact of multiple factors such as the structural adjustment of the real economy and the transformation of growth momentum under the new normal, it is increasingly difficult for farmers, especially those with low education level, to go out for employment, the difficulty of wage income growth and the risk of its contribution to the decline of farmers’ income are significantly increased (Zhang et al.,2020) [2]. How to realize the sustained growth of farmers’ income is a key topic for Chinese policy makers and researchers for a long time to come.

Capital is one of the important factors of production to increase farmers’ income. Many studies have found that financial development plays an important role in reducing poverty and increasing income (Jeanneney and Kpodar, 2011; Ding et al.,2022) [3,4]. Restraining financial development will increase the gap in economic development speed and income distribution between regions (Claessens and Feijen, 2006) [5]. Financial inclusion development can strengthen the role of finance in poverty reduction and income increase through financial empowerment and improving the availability of financial products and services (Miled and Rejeb, 2015) [6]. However, it should be noted that the complete satisfaction of farmers’ financing needs depends not only on the availability of financial products and services, but also on the residents’ own economic conditions and external economic conditions (Li and Li, 2020) [7]. From this point of view, easing the financial constraints may not be the potential mechanism of financial inclusion to promote the growth of farmers’ income. Compared with the direct supply of funds, financial inclusion is more fundamental and important for the improvement of rural production mode and productivity. For a long time, China’s rural areas have been carrying out the small-scale peasant production mode characterized by small production scale and low degree of division and cooperation. In this traditional mode of agricultural production, the effective demand for credit funds is insufficient, or farmers generally do not need to obtain funds from outside to maintain their original level and scale of production (Peng and Hu, 2018) [8]. By transforming the mode of production in rural areas and bringing farmers into the track of agricultural industrialization, financial inclusion can expand the use of farmers’ credit funds and improve their ability to use credit funds, which will increase the effective demand of farmers for credit funds. Therefore, the promotion of financial inclusion to agricultural industrialization may be an important channel for financial inclusion to increase farmers’ income.

In this theoretical and practical context, we use the provincial panel data from 2006 to 2022 to make an empirical analysis on the effect of financial inclusion on farmers’ income, and explore the transmission mechanism behind it. Compared with the previous literature, the contribution of this paper is reflected in the following aspects. First of all, although many scholars have studied the impact of financial inclusion on poverty reduction and income increase of farmers (Dupas and Robinson, 2013;Jiang and Liang, 2023) [9,10], but there is little in-depth analysis of the transmission mechanism of financial inclusion affecting farmers’ income increase from the perspective of agricultural industrialization. We study the impact of financial inclusion on farmers’ income, and empirically test the existence of the transmission mechanism of financial inclusion driving farmers’ income by promoting agricultural industrialization. Secondly, we find that the interaction between financial inclusion and agricultural industrialization has not promoted the growth of farmers’ income, which indicates that there are some structural problems in the development of financial inclusion in China. Through the division of labor and cooperation theory and development pole theory in economics, we further analyze the targeting mechanism of financial inclusion supporting farmers’ income. Thirdly, we analyzed the heterogeneity of financial inclusion in supporting farmers’ income increase from the perspective of income structure, income level, and regional characteristics, which can provide certain enlightenment and reference for formulating differentiated financial inclusion policies. Finally, we believe that financial inclusion should not only pay attention to the credit availability of disadvantaged groups (Von Fintel and Orthofer, 2020) [11], but also pay attention to the economic opportunities of disadvantaged groups and improve the ability of farmers to transform credit funds into productive capital.

The remainder of this paper is organized as follows. In Section 2, we put forward the research hypothesis of this paper on the basis of theoretical analysis. Our model, variables and data will be discussed in Section 3. We present the empirical results and analyzes them in Section 4. In Section 5, we further discuss the targeting mechanism of financial inclusion to support farmers’ income increase. We conclude and discuss the policy implications of our results in Section 6.

## Theoretical analysis and research hypothesis

### Financial inclusion and farmers’ income increase

The role of financial inclusion in increasing farmers’ income can be viewed from both direct and indirect aspects. The direct effect is reflected in the inclusion of marginal groups in the “traditional financial system” such as farmers into the financial service system by financial inclusion. Farmers can promote their own income by using financial products and services for investment, production and operation, smoothing consumption, resisting and diversifying risks. This includes improving the financial availability and lowering the barriers to obtain financial products and services of farmers by extending the spatial scope of financial products and services, expanding the reach of financial products and services.; By increasing the types of financial products and services and improving existing financial products and services, improve the effectiveness of farmers’ use of financial products and services; By reducing the cost of financial products and services, farmers’ willingness and ability to use financial products and services are improved, and so on. The indirect effect is mainly reflected in the increase of farmers’ income by promoting rural economic growth and improving the internal income distribution in rural areas. (1) Economic growth effect: Financial inclusion promotes economic development through functions such as mobilization of savings, capital accumulation, technological innovation, optimal allocation, and risk management. Economic development relies on the “trickle-down effect” to benefit farmers through the increase in the overall wealth level of the society and the rise in employment levels, thereby achieving farmers’ income growth and social welfare enhancement. (2) Income distribution effect: The lack of financial services is the cause of income inequality and even the poverty trap (Klapper et al., 2016) [12]. Financial inclusion makes up for the shortcomings of traditional finance that “dislike the poor and love the rich”. It promotes capital accumulation in rural areas with its financing function. And through the reallocation of funds, the threshold and cost for farmers to obtain financial products and services have been lowered, and farmers’ investment in human capital and other aspects has been increased, thereby improving farmers’ income levels and reducing income gaps.

### Financial inclusion and agricultural industrialization

Credit constraints are an important factor restricting the industrialization of agriculture and reducing agricultural performance. Under the traditional production mode, the accumulation of agricultural primitive capital is limited, and it is often unable to meet the capital needs of agricultural industrialization. However, the weak characteristics of long agricultural investment time, slow returns, and high risks have prompted financial institutions to reduce investment in rural areas for profitability purposes. Limited financial services in rural areas will restrict agricultural scale layout, crop selection, technology investment, and infrastructure construction (Conning and Udry, 2005) [13], hindering the transformation of rural areas from small-scale farming to agricultural industrialization. Financial inclusion can promote the development of agricultural industrialization through a variety of ways. On the one hand, financial inclusion can alleviate the credit constraints faced by agricultural business entities, and provide low-cost credit funds for agricultural business entities to introduce advanced technology and equipment, lease and transfer land, and hire agricultural labor to invest in rural industrial projects and expand production scale; It can also provide convenient and high-quality financial services for the upstream and downstream related agricultural business entities of the agricultural industry chain, which will help the extension of the agricultural industry chain. On the other hand, financial inclusion can play a guiding role, and the flow of credit funds will drive the flow and accumulation of production factors such as talents and technology to the field of agricultural industrialization. At the same time, it will create a good external environment for the development of agricultural industrialization by supporting rural infrastructure and public services such as roads, hydropower, warehousing, and logistics.

### Agricultural industrialization and farmers’ income increase

**Fig 1:**
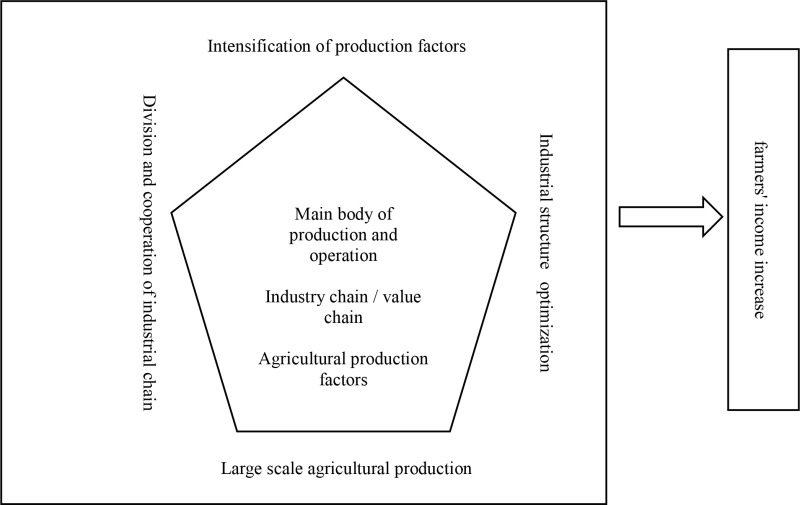
Theoretical framework of agricultural industrialization promoting farmers’ income increase.

China’s highly fragmented small-scale production methods have prevented the majority of farmers from improving agricultural production efficiency through large-scale operations (Hong, 2009) [14]. The majority of farmers engaged in traditional agriculture have achieved optimal allocation of production factors under resource constraints through continuous improvement of farming methods, but this optimal allocation can only solidify agricultural production in a low-end equilibrium state (Schultz, 1964) [15]. The industrialization of agriculture has broken the restriction of farmers’ traditional decentralized management methods on the further improvement of agricultural production efficiency. Through the application of agricultural machinery and equipment, agricultural science and technology in the production field, it has improved agricultural working conditions, expanded the scope of agricultural production operations, and formed the scale and specialization of agricultural production. Finally, the economic benefits of farmers engaged in agricultural production have been improved through economies of scale. Compared with non-agricultural industries, due to its own characteristics, the evolution of the division of labor in agriculture is relatively slow, the production is not very roundabout, and the industrial chain is relatively short (Cheng, 2012) [16]. Agricultural industrialization has brought about two effects through the extension of the agricultural industry chain. On the one hand, it integrates agricultural production, processing, sales and other links, which increases the roundaboutness of agricultural production and management, reduces the transaction costs of the internal links of agricultural production, and increases the added value of agricultural products, and promote the transfer of employment of farmers and the creation of income through entrepreneurship; On the other hand, it enables the majority of farmers to participate in the agricultural industry chain based on division of labor and cooperation, establishes the interest distribution mechanism between farmers and new industrial operators, closely combines the increase of farmers’ income with the development of rural industry, and ensures that farmers can reasonably share the value-added income of industrial development.The mechanism by which financial inclusion affects farmers’ income through agricultural industrialization is shown in Fig 1.

Based on the above analysis, this paper puts forward the following research hypotheses

H1: Financial inclusion can significantly promote the growth of farmers’ income.

H2: Financial inclusion can increase farmers’ income by promoting agricultural industrialization,

which is the transmission mechanism of financial inclusion to promote farmers’ income growth.

### Model, variable and data processing

#### Model setting.

According to the theoretical analysis and research hypothesis, we first test whether financial inclusion can effectively promote the growth of farmers’ income and set the following equation model:


$${Rev}_{i,t}=\alpha_{40}{+\alpha }_{41}{Ifi}_{i,t}+\gamma_{42}{Con}_{i,t}+\varepsilon_{4,i,t.}$$ (1)

Among them, the subscripts i and t correspond to regions and years respectively;${Rev}_{i,t}$,${Ifi}_{i,t}$ corresponding to farmers’ income and financial inclusion respectively; ${Con}_{i,t}$ is a series of control variables that may affect farmers’ income; $\varepsilon_{4,i,t}$ is random interference term.

Secondly, referring to the mediating effect test method proposed by Baron and Kenny (1986) [17], this paper introduces the mediating effect model to test whether agricultural industrialization is the transmission mechanism of financial inclusion promoting farmers’ income growth. We set the following equation model:


$${Rev}_{i,t}=\beta_{10}{+\beta }_{11}{Ifi}_{i,t}+\gamma_{12}{Con}_{i,t}+\varepsilon_{1,i,t},$$ (2)

$${Mid}_{i,t}=\beta_{20}{+\beta }_{21}{Ifi}_{i,t}+\gamma_{22}{Con}_{i,t}+\varepsilon_{2,i,t},$$ (3)

$${Rev}_{i,t}=\beta_{30}{+\beta }_{31}{Ifi}_{i,t}+\beta_{32}{Mid}_{i,t}+\gamma_{33}{Con}_{i,t}+\varepsilon_{3,i,t}.$$ (4)

Among them, ${Mid}_{i,t}$ is the intermediary variable of agricultural industrialization. The coefficient $\beta_{11}$ in equation (2) is the explanatory variable of the total effect of financial inclusion on farmers’ income; The coefficient $\beta_{21}$ in equation (3) is the effect of financial inclusion on the intermediary variables; In equation (4), coefficient $\beta_{31}$ is the influence of financial inclusion on farmers’ income after controlling the influence of intermediary variables, and $\beta_{32}$is the influence of intermediary variables on farmers’ income after controlling the influence of financial inclusion. When the coefficients $\beta_{11}$,$\beta_{21}$,$\beta_{32}$ in the equation are significant, there is a mediating effect, which indicates that financial inclusion can promote the growth of farmers’ income by promoting agricultural industrialization. The value of $\beta_{21}\beta_{32}$ represents the size of the mediating effect, and $\beta_{21}\beta_{32}$/$\beta_{11}$ represents the proportion of the mediating effect in the total effect, which is used to reflect the importance of mediating variables in the financial inclusion promoting the growth of farmers’ income. If the coefficient $\beta_{11}$ is significant and at least one of $\beta_{21}$and $\beta_{32}$is not significant, we need to further test the significance of the coefficient product (i.e. whether to reject H_0_: $\beta_{21}\beta_{32}$=0), and if it is significant, there is a mediating effect. When there is a mediating effect, if β_31 is not significant, it is a complete mediating effect, otherwise it is a partial mediating effect.

The intermediary effect model is used to verify whether financial inclusion can achieve the growth of farmers’ income by promoting agricultural industrialization, but it ignores the impact of agricultural industrialization on financial inclusion, that is, there may be interaction between financial inclusion and agricultural industrialization. Therefore, referring to the research of Chen (2023) [18], the interaction term of financial inclusion and agricultural industrialization is introduced on the basis of formula (4) to further examine the channel effect of financial inclusion on farmers’ income. The specific equation model is as follows:


$${Rev}_{i,t}=\alpha_{40}{+\alpha }_{41}{Ifi}_{i,t}+\alpha_{42}{Mid}_{i,t}+{\alpha_{43}Ifi}_{i,t}\times {Mid}_{i,t}+\gamma_{44}{Con}_{i,t}+\varepsilon_{4,i,t}.$$ (5)

### Variable definition

#### Farmers’ income.

Based on the practice of most existing literature, this paper uses the per capita income of rural residents to measure the growth of farmers’ income. Different from the existing literature, this paper further divides farmers’ income into wage income, family business income, property income and transfer income, which are expressed by Wrev,Frev,Prev,Trev respectively.

#### Financial inclusion.

Many studies measure financial inclusion by building an index. This construction is usually divided into four steps: index selection, index standardization, weight measurement and index synthesis. Reference to the relevant scholars’ research (Sarma and Pais., 2011;Xu et al., 2020) [19,20], we choose 10 indicators to form financial inclusion index system from three dimensions: accessibility of financial services, availability of financial services and utility of financial services. The accessibility of financial services is used to measure the penetration of financial services provided by a certain area among its users, that is, the extent to which residents in the area have access to or use financial services. The usage of financial services is used to measure the convenience of residents to obtain corresponding financial services in a certain area. The utility of financial services measures the actual utilization of financial services in a certain region and reflects the value of financial services for the whole society. Table 1 presents the specific contents of the indicators of financial inclusion.

**Table 1:** Financial inclusion indicators.

Dimension	Indicator	Definition	Properties
**Accessibility of financial services**	Branch accessibility	Number of bank branches/Area	Positive
		Number of bank branches/Population	Positive
	Staff accessibility	Number of bank staff/Area	Positive
		Number of bank staff/Population	Positive
**Usage of financial services**	Savings per capita	Savings/Population	Positive
	Loans per capita	Loans/Population	Positive
	Insurance density	Insurance revenue/Population	Positive
**Utility of financial services**	Savings ratio	Savings/GDP	Positive
	Loans ratio	Loans/GDP	Positive
	Insurance depth	Insurance revenue/GDP	Positive

Due to the different units of the original financial inclusive indicators, before we calculate the financial inclusion index, we need to standardize the indicators. After the standardization, the value range of each indicator is [0,1]. Since the selected indicators are all positive indicators, the standardization method is shown in formula (6):


$$\begin{array}{c} x_{ij}=\frac{A_{ij-m_{ij}}}{M_{ij-m_{ij}}} \end{array}$$ (6)

Among them, i represents the i-th dimension, j represents the j-th indicator under this dimension, $x_{ij}$ represents the indicator value after standardization, $A_{ij}$ represents the original indicator value before standardization, $m_{ij}$ and $M_{ij}$ represent the minimum and maximum value of the original indicator respectively.

We use the coefficient of variation method to determine the weight of each index in its dimension and the weight of each dimension. The basic principle is that the more information or the greater the degree of numerical dispersion of an index, the greater the weight value will be given. The weight calculated by this method is more objective, which makes the final index more accurate. Many scholars use this method to calculate the financial inclusion index. The weight calculation method of each index or dimension is shown in formula (7):


$$\begin{array}{c} w_{i}=\frac{V_{i}}{\sum_{i=1}^{n}V_{i}}, \end{array}$$ (7)

where $w_{i}$ is the weight of the i-th indicator or dimension, $V_{i}$ is the coefficient of variation of the i-th indicator or dimension, and the calculation method of $V_{i}$ is shown in formula (8):


$$\begin{array}{c} V_{i}=\frac{S_{i}}{A_{i}} \end{array}$$ (8)

where $S_{i}$ is the standard deviation of the i-th indicator or dimension, and $A_{i}$ is the average of the i-th indicator or dimension.

Financial inclusion index synthesis mainly includes dimension value synthesis and financial inclusion index synthesis, which are obtained by measuring the European distance between the standardized value and ideal value 1 of specific indicators in each dimension. The specific method of dimension value measurement is shown in formula (9):


$$\begin{array}{c} {Ifi}_{i}=1-\frac{\sqrt{w_{i1}^{2}{\left(1-x_{i1}\right)}^{2}+w_{i2}^{2}{\left(1-x_{i2}\right)}^{2}+\ldots +w_{ij}^{2}{\left(1-x_{ij}\right)}^{2}}}{\sqrt{\left(w_{i1}^{2}+w_{i2}^{2}+\ldots w_{ij}^{2}\right)}} \end{array}$$ (9)

After calculating the value of each dimension by formula (9), we use formula (10) to further synthesize the financial inclusion index. The specific method is shown in formula (10):


$$\begin{array}{c} Ifi=1-\frac{\sqrt{w_{1}^{2}{\left(1-{IFI}_{1}\right)}^{2}+w_{2}^{2}{\left(1-{IFI}_{2}\right)}^{2}+w_{3}^{2}{\left(1-{IFI}_{3}\right)}^{2}}}{\sqrt{w_{1}^{2}+w_{2}^{2}+w_{3}^{2}}}. \end{array}$$ (10)

Among them, ${Ifi}_{1}$, ${Ifi}_{2}$ and ${Ifi}_{3}$ represent the three dimensions of financial inclusion, such as accessibility of financial services, usage of financial services and utility of financial services.

### Agricultural industrialization (Agri)

The essence of agricultural industrialization lies in the transformation of agricultural production and management mode from traditional agriculture to modern agriculture, and efforts to promote agricultural industrialization. Referring to the research of relevant scholars (Peng and Xu, 2019; Chen, 2020) [21,22], this paper intends to measure the development level of agricultural industrialization from four aspects: structuration (Ais), large-scale (Apl), intensification (Api), and integration (Ahl), and use the above method to build the agricultural industrialization index. Among them, structuration is used to measure the degree of adjustment and optimization of agricultural production structure. Large-scale is used to measure the scale of production and operation in the process of agricultural industrialization. Intensification is used to measure the rational allocation of agricultural production factors such as talents, science and technology. Integration is used to measure the division of labor and cooperation within the agricultural industry system and the extension of the agricultural industry chain. Table 2 reports the specific contents of agricultural industrialization indicators.

**Table 2:** Indicators of agricultural industrialization.

Dimension	Indicator	Definition	Properties
**Structuration**	Agricultural output ratio	Agricultural output/ Agriculture, forestry, animal husbandry and fishery output	Positive
**Intensification**	Agricultural machinery power per unit area	Machinery power of agriculture/ Sown area of crops	Positive
	Agriculture, forestry, animal husbandry and fishery output per capita	Agriculture, forestry, animal husbandry and fishery output/Rural population	Positive
**Integration**	Agriculture, forestry, animal husbandry and fishery service industry output ratio	Agriculture, forestry, animal husbandry and fishery service industry output/Agriculture, forestry, animal husbandry and fishery output	Positive
**Large-scale**	Sown area of crops per capita	Sown area of crops/ Number of employees in the primary industry	Positive
	Agricultural fixed assets investment per capita	Agricultural fixed assets investment/ Number of employees in the primary industry	Positive

### Control variables

In order to eliminate the influence of other factors on the results, this paper takes the relevant factors that have an important impact on farmers’ income as control variables into the econometric model, which are industrial structure, population density, government economic participation, economic openness, and transportation development. (1) Industrial structure (Ids). The upgrading of industrial structure will promote the transfer of rural surplus labor from agriculture to secondary and tertiary industries, which will have an important impact on the income level of farmers. This paper uses the proportion of the added value of the second and third industries in the GDP of each region to reflect the industrial structure. (2) Population density (Pde). Population density is an important index to measure the distribution of population in the region, which is closely related to resources and economy. This paper uses the population quantity of unit land area to measure the population density of each region. (3)Government economic participation (Gep).The government can control the investment scale and direction of public financial resources to affect the allocation efficiency of regions. This paper uses the proportion of fiscal expenditure in GDP as an indicator of government economic participation. (4)Economic openness (Ope). Foreign trade can improve the welfare of the poor through consumer surplus channels, and also provide employment opportunities for them through producer surplus channels (Muhammad et al., 2010) [23]. This paper uses the proportion of total foreign investment in GDP to express economic openness. (5)Transportation development (Inf). Traffic road construction can produce spatial spillover effect and Tiebout mechanism, which will have an important impact on the economic development of rural areas. This paper uses the highway mileage per unit of land area to measure the traffic development level of each region.

### Data sources

In order to compare the sample data and take into account the integrity and availability of data, this paper selects panel data from 30 provincial administrative regions of Chinese mainland from 2006 to 2022 to conduct an empirical analysis. The data in Tibet area is missing seriously, so the study sample will be excluded. The original data of financial inclusion indicators come from China Financial Yearbook and regional financial operation reports. The original data of other variables are from China Statistical Yearbook, China Rural Statistical Yearbook, China Industrial statistical yearbook and statistical yearbooks of provincial administrative regions.

### Descriptive statistics

Table 3 reports the descriptive statistical results of the main variables during the sample period. (1) The average value of farmers’ income is 0.0113, the minimum value is 0.0020, but the maximum value is as high as 0.0397. There are large regional differences in farmers’ income levels. The average values of wage income, family operation income, property income and transfer income are 0.0050, 0.0041, 0.0004 and 0.0019, which indicates that wage income and family operating income are the main sources of farmers’ income. (2) The average value of the financial inclusion index is 0.1130. Considering that the value range of the index is between 0 and 1, it shows that the development level of financial inclusion of the provinces in the sample is generally low to a certain extent. There is also a large gap between the maximum and minimum value of financial inclusion index, which indicates that there is a large regional imbalance in the development level of financial inclusion in the 30 provinces in the sample. Among them, the utility index has the largest mean value of 0.2473, which is 2.6 times of the average value of the accessibility index (0.0959), and the usage index has the middle mean value of 0.1196.(3) The average value of the agricultural industrialization index is 0.1499, indicating that the overall level of agricultural industrialization in China is relatively low. However, from 2006 to 2022, the level of agricultural industrialization showed a steady upward trend. The mean values of structuration, large-scale, intensification, and integration are 0.4598, 0.0708, 0.2689 and 0.2473 respectively. Due to space limitations, we will not repeat the statistical characteristics of the control variables.

**Table 3:** Descriptive statistics.

Variables	Number of observations	Mean value	Standard deviation	Minimum value	Maximum
**Rev**	510	0.0113	0.0068	0.0020	0.0397
**Wrev**	510	0.0050	0.0044	0.0003	0.0250
**Frev**	510	0.0041	0.0020	0.0006	0.0111
**Prev**	510	0.0004	0.0004	0.00002	0.0036
**Trev**	510	0.0019	0.0017	0.0001	0.0115
**Ifi**	510	0.1130	0.1213	0.0178	0.8144
**Ifi**_**1**_	510	0.0959	0.1459	0.0091	0.9166
**Ifi**_**2**_	510	0.1196	0.1305	0	1
**Ifi**_**3**_	510	0.2473	0.1465	0.0164	0.8692
**Agri**	510	0.1499	0.0514	0.0409	0.4573
**Ais**	510	0.4598	0.2102	0	1
**Apl**	510	0.0708	0.0778	0.0074	0.7375
**Api**	510	0.2689	0.1217	0.0190	0.7101
**Ahl**	510	0.2473	0.1615	0	1
**Ids**	510	0.8968	0.0554	0.6728	0.9978
**Pde**	510	0.0457	0.0678	0.0008	0.3926
**Gep**	510	0.2313	0.0986	0.0830	0.6430
**Ope**	510	0.7178	3.5268	0.0482	55.9380
**Inf**	510	0.9080	0.4979	0.0661	2.2589

### Empirical results and analysis

#### Measurement results of financial inclusion index.

Table 4 specifically reports the average value of financial inclusion and its sub indexes in 30 provinces of China from 2006 to 2022. In the sample period, the average value of financial inclusion index in the eastern, central and western regions is 0.1962, 0.0697 and 0.0613 respectively, which indicates that the development level of financial inclusion in the eastern coastal areas is higher than that in the central and western regions on the whole, showing a decreasing trend from east to west, while the gap between the development level of financial inclusion in the western and central regions is small on the whole. Specifically, Shanghai’s average value of financial inclusion is the highest, which is 0.5886, while Guangxi’s average value of financial inclusion is the lowest, which is 0.0459, showing that the development level of financial inclusion between provinces presents difference. Although Hebei is adjacent to Beijing and Tianjin where the development level of financial inclusion is relatively high, it has not been greatly affected by the driving effect, and the level of financial inclusion is relatively low in the eastern region. Although Chongqing is in the western region with the lowest average value of financial inclusion, the level of financial inclusion in Chongqing is higher than that in other western regions, and also higher than that in Fujian, Hainan and Hebei provinces in the eastern region. As far as the sub indexes of financial inclusion are concerned, the average values of accessibility, usage and utility are still the highest in the eastern region. The average accessibility of the central region is higher than that of the western region, while the average usage and utility of the western region is higher than that of the central region. Shanghai had the highest average accessibility (0.7666), while Beijing had the highest usage (0.5387) and utility (0.7587). Xinjiang, Guangxi and Inner Mongolia are the regions with the lowest mean values of accessibility, usage and utility respectively, which are all located in the western region.

**Table 4:** Average value of financial inclusion index of 30 provinces.

Region	Financial inclusion	Accessibility	Usage	Utility
**Eastern Region**				
**Beijing**	0.4087	0.3336	0.5387	0.7587
**Tianjin**	0.2644	0.2831	0.2292	0.2746
**Hebei**	0.0813	0.0655	0.0778	0.2154
**Liaoning**	0.1082	0.0838	0.1163	0.2656
**Shanghai**	0.5886	0.7666	0.3979	0.5231
**Jiangsu**	0.1452	0.1300	0.1640	0.1940
**Zhejiang**	0.1660	0.1374	0.1863	0.3123
**Fujian**	0.0869	0.0651	0.1121	0.1555
**Shandong**	0.0981	0.0963	0.0926	0.1324
**Guangdong**	0.1338	0.1094	0.1467	0.2727
**Hainan**	0.0772	0.0512	0.0817	0.2573
**Eastern average**	0.1962	0.1929	0.1948	0.3056
**Central region**				
**Shanxi**	0.0892	0.0590	0.0935	0.3086
**Jilin**	0.0718	0.0487	0.0858	0.1984
**Heilongjiang**	0.0596	0.0321	0.0755	0.2130
**Anhui**	0.0723	0.0582	0.0685	0.1897
**Jiangxi**	0.0621	0.0461	0.0647	0.1702
**Henan**	0.0759	0.0762	0.0592	0.1352
**Hubei**	0.0695	0.0476	0.0867	0.1668
**Hunan**	0.0571	0.0466	0.0609	0.1187
**Central average**	0.0697	0.0518	0.0744	0.1876
**Western Region**				
**Inner Mongolia**	0.0527	0.0264	0.0917	0.1062
**Guangxi**	0.0459	0.0301	0.0492	0.1482
**Chongqing**	0.0900	0.0595	0.1068	0.2596
**Sichuan**	0.0700	0.0392	0.0809	0.2641
**Guizhou**	0.0516	0.0294	0.0506	0.2201
**Yunnan**	0.0472	0.0173	0.0566	0.2368
**Shaanxi**	0.0727	0.0437	0.0905	0.2231
**Gansu**	0.0586	0.0234	0.0647	0.3178
**Qinghai**	0.0584	0.0188	0.0854	0.2629
**Ningxia**	0.0731	0.0379	0.0921	0.2714
**Xinjiang**	0.0543	0.0152	0.0823	0.2479
**Western average**	0.0613	0.0310	0.0773	0.2326

**Fig 2:**
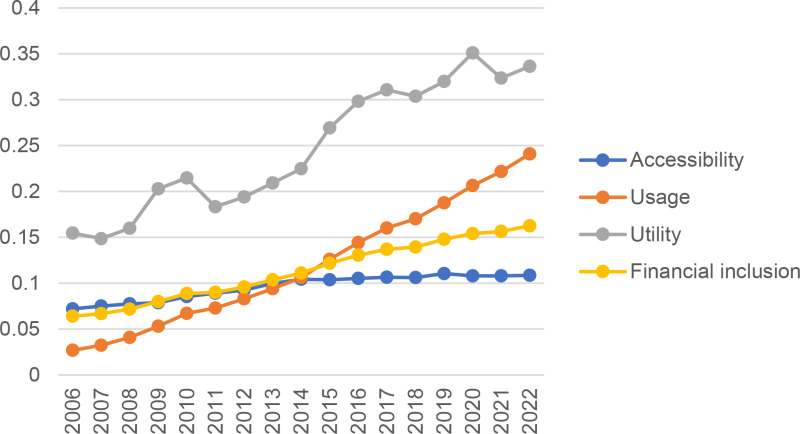
Time trend of financial inclusion and its sub index.

Fig 2 depicts the trend of financial inclusion and its sub index from 2006 to 2022. The financial inclusion and its sub index accessibility and usage show a slow rising trend in the sample period, and the rising range is in the order of usage, financial inclusion and accessibility from large to small. There is no significant difference in financial inclusion, accessibility and usage. The utility showed a fluctuating upward trend during the sample period. Although the utility had a slight downward trend from 2010 to 2011, it was gradually adjusted in the later period, and the utility level was far greater than the level of financial inclusion and its accessibility and usage.

#### Basic regression results.

Firstly, we use equation (1) to investigate the effect of financial inclusion on farmers’ income under the whole sample. The regression results are shown in Table 5. The impact of financial inclusion on farmers’ income is significantly positive, and has passed the 1% significance level test, which shows that financial inclusion can significantly promote the growth of farmers’ income. Considering that different dimensions of financial inclusion may have different effects on farmers’ income, we further test the impact of accessibility, usage and utility on farmers’ income. Accessibility, usage and utility have significant positive effects on the growth of farmers’ income, and pass the significant level of 1%. This shows that with the increase of the accessibility, usage and utility of financial inclusion, the access requirements of financial services for rural residents are reduced, so that more people can enjoy the convenience and welfare brought by financial services, which is conducive to the income growth of rural residents. In terms of impact effect, accessibility (0.0662) and usage (0.0528) have relatively greater impact on farmers’ income, while utility (0.0439) has relatively less impact on farmers’ income. Furthermore, in column (5), we also examine the effects of accessibility, usage and utility on farmers’ income simultaneously. Usage and utility still have a significant positive impact on farmers’ income, while accessibility no longer has a significant impact on farmers’ income, which indicates that there are certain differences in the effects of different dimensions of financial inclusion on farmers’ income. In the control variables, the coefficients of industrial structure and transportation development are significantly positive at the 1% level respectively, and both of them have a significant positive effect on the increase of rural residents’ income. The upgrading of industrial structure can provide non-agricultural employment opportunities for the transferred agricultural labor force, which is conducive to the growth of rural residents’ income (Wen and Wang, 2015) [24]. The improvement of transportation development level will promote the division of labor of farmers, improve production efficiency, and is conducive to the growth of rural residents’ income. The coefficient of population density is significantly negative at the level of 1%, and population density has a significant negative effect on the increase of farmers’ income. This shows that China’s demographic dividend gradually disappears with the continuous increase of population density, its marginal effect on economic growth decreases, and it has a negative effect on the growth of rural residents’ income. Government economic participation and economic openness have a positive effect on the growth of farmers’ income, but there is no significant correlation.

**Table 5:** Basic regression results.

Variable	Rev				
	(1)	(2)	(3)	(4)	(5)
**Ifi**	0.1337*** (0.0039)				
**Ifi**_**1**_		0.0662*** (0.0133)			−0.0025 (0.0068)
**Ifi**_**2**_			0.0528*** (0.0015)		0.0451*** (0.0016)
**Ifi**_**3**_				0.0439*** (0.0025)	0.0164*** (0.0019)
**Ids**	0.0181*** (0.0054)	0.0225** (0.0098)	0.0135*** (0.0052)	0.0423*** (0.0079)	0.0220*** (0.0049)
**Pde**	−0.3333*** (0.0232)	−0.0381 (0.0667)	−0.0746*** (0.0173)	0.2155*** (0.0219)	−0.0323 (0.0339)
**Gep**	0.0003 (0.0023)	0.0293*** (0.0038)	0.0125*** (0.0021)	−0.0213*** (0.0043)	−0.0044 (0.0027)
**Ope**	0.0000 (0.0000)	−0.0000 (0.0000)	0.0000 (0.0000)	0.0000 (0.0000)	0.0000 (0.0000)
**Inf**	0.0100*** (0.0009)	0.0215*** (0.0015)	0.0104*** (0.0009)	0.0115*** (0.0014)	0.0082*** (0.0009)
**_cons**	−0.0139*** (0.0046)	−0.0398*** (0.0083)	−0.0161*** (0.0044)	−0.0528*** (0.0065)	−0.0226*** (0.0043)
**Id**	30	30	30	30	30
**Obs**	510	510	510	510	510
**R-sq**	0.9058	0.6930	0.9126	0.8038	0.9249

Note: ***, ** and * represent significance levels of 1%, 5% and 10%, respectively. Standard errors are indicated in brackets, and the following table is the same.

### Heterogeneity analysis

#### Analysis on the heterogeneity of income structure.

In order to further explore the impact of financial inclusion on the heterogeneity of farmers’ income increase in different income structures, we further decompose farmers’ income into wage income, family business income, property income and transfer income. Table 6 reports the regression results of the heterogeneity of income structure. Financial inclusion can significantly promote the growth of farmers’ wage income, family operating income, property income and transfer income, and has passed the 1% significance level test. The regression coefficients of financial inclusion to wage income, family operating income, property income and transfer income are 0.0671, 0.0264, 0.0043 and 0.0359 respectively, which shows that the promotion of financial inclusion to wage income is significantly stronger than that of family operating income. The possible reason is that the traditional small-scale agricultural production mode is still the main mode in rural areas of China at present, the level of agricultural industrialization is relatively low, the demand for farmers to use financial capital to serve agricultural production and operation is limited, which leads to the influence of financial inclusion on the income of farmers’ family operation is weaker than that of wage income.

**Table 6:** Regression results of income structure heterogeneity.

Variable	Wrev	Frev	Prev	Trev
**Ifi**	0.0671*** (0.0024)	0.0264*** (0.0018)	0.0043*** (0.0004)	0.0359*** (0.0014)
**Controls**	Yes	Yes	Yes	Yes
**Id**	30	30	30	30
**Obs**	510	510	510	510
**R-sq**	0.8612	0.7449	0.4361	0.8379

### Analysis on the heterogeneity of income level

In order to further explore the heterogeneous impact of financial inclusion on the income increase of farmers with different income levels, we divided the research samples into 10%, 25%, 50%, 75% and 90% quantiles according to the income level, corresponding to the lowest income group, middle and low income group, middle income group, middle and high income group and the highest income group respectively, then panel quantile regression is performed on the research sample, and the heterogeneity regression results of income level are reported in Table 7. The regression results show that financial inclusion can significantly promote the growth of farmers’ income in different sub groups, and the influence coefficients are 0.1127, 0.1202, 0.1310, 0.1452 and 0.1585 respectively. This shows that the effect of financial inclusion on the low-end group of farmers’ income is significantly less than that on the middle and high-end groups. The effect of financial inclusion on the income of farmers of different income classes is heterogeneous. The reason is that the low-income farmers are constrained by more internal and external conditions, such as physical capital, human capital, geographical environment, economic development environment and so on. As a result, they lack the opportunity and ability to use financial capital to obtain factor input returns and realize their own development, weakening the impact of financial inclusion on the low-income farmers’ income.

**Table 7:** Regression results of income level heterogeneity.

Variable	Rev				
	10% quantile	25% quantile	50% quantile	75% quantile	90% quantile
**Ifi**	0.1127*** (0.0123)	0.1202*** (0.0094)	0.1310*** (0.0074)	0.1452*** (0.0103)	0.1585*** (0.0158)
**Controls**	Yes	Yes	Yes	Yes	Yes
**Id**	30	30	30	30	30
**Obs**	510	510	510	510	510

### Analysis of regional heterogeneity

According to the differences of infrastructure and information level in different regions, the research samples were divided into two groups for regional heterogeneity analysis. Columns (1) and (2) of Table 8 are the analysis of regional heterogeneity by dividing the study samples into two categories: higher infrastructure and lower infrastructure. We refer to Zou (2019) [25] and use the level of financial support for agriculture as an alternative variable of infrastructure. The level of financial support for agriculture is measured by the proportion of local agricultural, forestry and water affairs expenditure in financial expenditure. From 2006 to 2022, the infrastructure variables of each region were ranked from high to low, and divided into two groups according to the average size. The regression results show that financial inclusion has a more significant effect on the income of rural residents with higher infrastructure. The possible explanation for this is that on the one hand, the development of infrastructure can attract all kinds of production factors including financial capital to gather in rural areas, help rural residents more easily obtain credit support, and ease their liquidity constraints; On the other hand, it can improve the local economic development environment and create more economic opportunities for rural residents to use financial capital. In columns (3) and (4) of Table 8, the research samples are divided into two categories: high information level and low information level for regional heterogeneity analysis. We use the ratio of the total amount of post and telecommunications business in each region to GDP to measure the information level. Similarly, the information level variables of each region from 2006 to 2022 were ranked from high to low, and divided into two groups according to the average size. The regression results show that financial inclusion has a more significant effect on the income of farmers with higher information level. This may be because information asymmetry is an important factor in credit rationing, and the cost of commercial banks to obtain customer information is relatively high in rural areas due to the relatively backward communication facilities. The improvement of information level will ease the information asymmetry between commercial banks and customers, and help to play the role of financial inclusion in promoting farmers’ income growth.

**Table 8:** Regression results of regional heterogeneity.

Variable	Rev			
	High infrastructure (1)	Low infrastructure (2)	High level of information (3)	Low level of information (4)
**Ifi**	0.1971*** (0.0059)	0.1128*** (0.0058)	0.1905*** (0.0062)	0.1192*** (0.0048)
**Controls**	Yes	Yes	Yes	Yes
**Id**	15	15	15	15
**Obs**	255	255	255	255
**R-sq**	0.9509	0.9083	0.9514	0.9270

### Mechanism test

#### Analysis of mediating effect.

In order to deeply analyze whether agricultural industrialization is the transmission mechanism of financial inclusion promoting farmers’ income growth, we use the intermediary effect to test the agricultural industrialization mechanism, and report the regression results in Table 9. In step 1, financial inclusion has a significant positive impact on farmers’ income growth; In step 2, financial inclusion has a significant positive impact on agricultural industrialization; In step 3, financial inclusion and agricultural industrialization have a significant positive impact on farmers’ income growth, and compared with step 1, the impact coefficient of financial inclusion on farmers’ income growth becomes smaller after the introduction of intermediary variables. This shows that financial inclusion can indirectly promote the growth of farmers’ income by promoting agricultural industrialization, and agricultural industrialization plays a part of intermediary effect in it. After using the same method to analyze the four dimensions of agricultural industrialization, it is found that intensification and integration are indirect mechanisms for financial inclusion to promote farmers’ income growth, and intensification and integration play a part of intermediary effect. However, structuration and large-scale do not have a mediating effect in the process of financial inclusion promoting farmers’ income growth. This indicates that financial inclusion mainly promotes the development of agricultural industrialization through agricultural intensification and integration, thereby driving the growth of farmers’ income. The total effect of financial inclusion on farmers’ income is 0.1337, and the indirect effect of agricultural industrialization is 0.0023. Agricultural industrialization can explain 1.7432% of the total effect of financial inclusion on farmers’ income. We further compared the proportion of the indirect effects of the dimensions of agricultural industrialization in the total effects. The indirect effects of intensification and integration were 0.0182 and 0.0024, accounting for 13.6341% and 1.8223% of the total effects.

**Table 9:** Regression results of mediating effect.

Variable	Step1	The intermediary effect of industrialization		The intermediary effec structural		The intermediary effec large scale		The intermediary effec intensive		The mediating effect of integration	
		Step2	Step3	Step2	Step3	Step2	Step3	Step2	Step3	Step2	Step3
	Rev	Agri	Rev	Ais	Rev	Apl	Rev	Api	Rev	Ahl	Rev
**Ifi**	0.1337*** (0.0039)	0.2285*** (0.0651)	0.1313*** (0.0039)	−0.1563 (0.1525)	0.1336*** (0.0039)	−0.1139 (0.1045)	0.1339*** (0.0039)	0.9907*** (0.1206)	0.1154*** (0.0035)	1.2185*** (0.1515)	0.1312*** (0.0042)
**Agri**			0.0102*** (0.0027)								
**Ais**					−0.0002 (0.0012)						
**Apl**							0.0025 (0.0017)				
**Api**									0.0184*** (0.0012)		
**Ahl**											0.0020* (0.0012)
**Controls**	Yes	Yes	Yes	Yes	Yes	Yes	Yes	Yes	Yes	Yes	Yes
**Id**	30	30	30	30	30	30	30	30	30	30	30
**Obs**	510	510	510	510	510	510	510	510	510	510	510
**R-sq**	0.9058	0.4480	0.9084	0.1148	0.9058	0.4749	0.9062	0.6853	0.9357	0.3420	0.9063

#### Analysis of interaction effect.

Considering the possible interaction between financial inclusion and agricultural industrialization, we further introduce the interaction term between financial inclusion and agricultural industrialization into the model, and the regression results of interaction effect are reported in Table 10. The estimated coefficients of financial inclusion and agricultural industrialization are significantly positive, while the interaction coefficient between financial inclusion and agricultural industrialization is not significant, reflecting that the improvement of financial inclusion level has not enhanced the marginal effect of agricultural industrialization on farmers’ income increase. This means that financial inclusion has not become a positive factor for agricultural industrialization to promote farmers’ income. In order to explore the reasons for the insignificant interaction coefficient between financial inclusion and agricultural industrialization, we further analyzed the interaction effect of four sub dimensions of financial inclusion and agricultural industrialization. The regression results show that the estimated coefficient of financial inclusion is positive, the estimated coefficients of intensification and integration are positive, the interaction coefficient of financial inclusion with intensification is positive, and the interaction coefficient with integration is negative. This reflects that the improvement of financial inclusion increases the marginal effect of agricultural intensification on farmers’ income, but reduces the marginal effect of agricultural integration on farmers’ income. This shows that the reason why financial inclusion has not become a positive factor affecting agricultural industrialization to promote farmers’ income increase may be that financial inclusion has effectively played a supporting role in agricultural intensification agricultural scale, but the support for agricultural integration needs to be improved. There are some structural problems in China’s current financial inclusion development model, so we need to change its development model in order to better play its role in supporting agricultural industrialization and driving farmers’ income.

**Table 10:** Regression results of interaction effect.

Variable	Rev				
**Ifi**	0.1302*** (0.0046)	0.1334*** (0.0053)	0.1310*** (0.0039)	0.0879*** (0.0046)	0.1567*** (0.0051)
**Agri**	0.0085* (0.0044)				
**Ais**		−0.0003 (0.0015)			
**Apl**			−0.0092*** (0.0031)		
**Api**				0.0102*** (0.0015)	
**Ahl**					0.0070*** (0.0013)
**Ifi×Agri**	0.0067 (0.0136)				
**Ifi × Ais**		0.0006 (0.0087)			
**Ifi × Apl**			0.0367*** (0.0081)		
**Ifi × Api**				0.0950*** (0.0111)	
**Ifi × Ahl**					−0.0418*** (0.0053)
**Controls**	Yes	Yes	Yes	Yes	Yes
**Id**	30	30	30	30	30
**Obs**	510	510	510	510	510
**R-sq**	0.9085	0.9058	0.9101	0.9443	0.9174

### Endogeneity and robustness test

In order to reduce the interference of endogenous problems caused by mutual causality of variables on the research conclusion, we conduct first-order lag on all explanatory variables and control variables, and use entropy method to remeasure the financial inclusion index for empirical test. The calculation steps of entropy method refer to the research of Liu et al(2021) [26]. Table 11 shows the endogenous and robust test results of the basic regression. Financial inclusion and its three sub dimensions of accessibility, usage and utility have a significant positive impact on farmers’ income, and pass the 1% significance level test, which shows that the conclusion that financial inclusion can effectively promote farmers’ income growth is robust. We also performed endogenous and robust tests for heterogeneity regression, and the corresponding regression results are reported in Tables 12–14. Financial inclusion can significantly promote the growth of farmers’ wage income, family business income, property income and transfer income; The promoting effect of financial inclusion on farmers’ income increases with the increase of farmers’ income level; Financial inclusion plays a stronger role in promoting farmers’ income in areas with higher infrastructure and information level, which is consistent with the research conclusion of the original regression. In addition, we also test the endogenous and robust transmission mechanism of financial inclusion promoting farmers’ income growth. The results of endogenous and robust tests for mediating effect and interaction effect regression are reported in Tables 15 and 16 respectively. Agricultural industrialization is the transmission mechanism of financial inclusion to promote farmers’ income growth, but the current financial inclusion has not become a positive factor of agricultural industrialization to promote farmers’ income growth, and the regression results have not changed the original regression conclusions.

**Table 11:** Endogeneity and robustness test: Basic regression results.

Variable	Rev				
	(1)	(2)	(3)	(4)	(5)
**Ifi**	6.7589*** (0.2653)				
**Ifi**_**1**_		1.6656*** (0.5060)			−0.0419 (0.2703)
**Ifi**_**2**_			3.2544*** (0.0996)		2.8647*** (0.1109)
**Ifi**_**3**_				4.6537*** (0.3128)	1.5878*** (0.2326)
**Ids**	0.0224*** (0.0061)	0.0270*** (0.0094)	0.0173*** (0.0052)	0.0428*** (0.0078)	0.0237*** (0.0050)
**Pde**	−0.3613*** (0.0293)	0.0790 (0.0596)	−0.0623*** (0.0171)	0.2101*** (0.0214)	−0.0355 (0.0323)
**Gep**	0.0104*** (0.0026)	0.0353*** (0.0037)	0.0148*** (0.0021)	−0.0095** (0.0043)	0.0017 (0.0028)
**Ope**	−0.0000 (0.0000)	−0.0000 (0.0001)	0.0000 (0.0000)	0.0000 (0.0000)	0.0000 (0.0000)
**Inf**	0.0124*** (0.0010)	0.0208*** (0.0015)	0.0104*** (0.0009)	0.0130*** (0.0014)	0.0090*** (0.0009)
**_cons**	−0.0182*** (0.0052)	−0.0461*** (0.0079)	−0.0196*** (0.0044)	−0.0545*** (0.0064)	−0.0246*** (0.0043)
**Id**	30	30	30	30	30
**Obs**	480	480	480	480	480
**R-sq**	0.8852	0.7241	0.9170	0.8114	0.9250

**Table 12:** Endogeneity and robustness test: Regression results of income structure heterogeneity.

Variable	Wrev	Frev	Prev	Trev
**Ifi**	3.4024*** (0.1564)	1.3131*** (0.1107)	0.2897*** (0.0227)	1.7538*** (0.0972)
**Controls**	Yes	Yes	Yes	Yes
**Id**	30	30	30	30
**Obs**	480	480	480	480
**R-sq**	0.8419	0.7454	0.4589	0.7962

**Table 13:** Endogeneity and robustness test: Regression results of income level heterogeneity.

Variable	Rev				
	10% quantile	25% quantile	50% quantile	75% quantile	90% quantile
**Ifi**	5.9933*** (0.6275)	6.2865*** (0.4651)	6.6780*** (0.3764)	7.1687*** (0.5551)	7.6782*** (0.8818)
**Controls**	Yes	Yes	Yes	Yes	Yes
**Id**	30	30	30	30	30
**Obs**	480	480	480	480	480

**Table 14:** Endogeneity and robustness test: Regression results of regional heterogeneity.

Variable	Rev			
	High infrastructure (1)	Low infrastructure (2)	High level of information (3)	Low level of information (4)
**Ifi**	11.4268*** (0.3863)	5.3923*** (0.3823)	10.6783*** (0.3901)	5.9005*** (0.3205)
**Controls**	Yes	Yes	Yes	Yes
**Id**	15	15	15	15
**Obs**	240	240	240	240
**R-sq**	0.9473	0.8894	0.9496	0.9149

**Table 15:** Endogeneity and robustness test: Regression results of mediating effect.

Variable	Step1	The mediating effect of industrialization		The mediating effect of structure		The mediating effect of large scale		The mediating effect of intensification		The mediating effect of integrated	
		Step2	Step3	Step2	Step3	Step2	Step3	Step2	Step3	Step2	Step3
	Rev	Agri	Rev	Ais	Rev	Apl	Rev	Api	Rev	Ahl	Rev
**Ifi**	6.7589*** (0.2653)	17.6712*** (3.9410)	6.6712*** (0.2626)	−8.2445 (9.4328)	6.7508*** (0.2673)	3.4891 (6.2577)	6.7930*** (0.2660)	44.7814*** (7.2881)	5.8599*** (0.2345)	62.0525*** (9.3777)	6.4787*** (0.2771)
**Agri**			0.0110*** (0.0030)								
**Ais**					−0.0004 (0.0013)						
**Apl**							0.0027 (0.0019)				
**Api**									0.0197*** (0.0015)		
**Ahl**											0.0043*** (0.0013)
**Controls**	Yes	Yes	Yes	Yes	Yes	Yes	Yes	Yes	Yes	Yes	Yes
**Id**	30	30	30	30	30	30	30	30	30	30	30
**Obs**	480	480	480	480	480	480	480	480	480	480	480
**R-sq**	0.8852	0.4817	0.8887	0.1002	0.8852	0.5245	0.8858	0.6780	0.9180	0.3457	0.8878

**Table 16:** Endogeneity and robustness test: Regression results of interaction effect.

Variable	Rev				
**Ifi**	6.3878*** (0.2812)	6.7793*** (0.3103)	6.6572*** (0.2609)	3.9177*** (0.3184)	6.8368*** (0.3233)
**Agri**	0.0018 (0.0045)				
**Ais**		−0.0002 (0.0016)			
**Apl**			−0.0095*** (0.0024)		
**Api**				0.0118*** (0.0017)	
**Ahl**					0.0057*** (0.0015)
**Ifi×Agri**	1.8902*** (0.7012)				
**Ifi × Ais**		−0.0986 (0.5439)			
**Ifi × Apl**			1.9945*** (0.4081)		
**Ifi × Api**				6.2018*** (0.7407)	
**Ifi × Ahl**					−0.6312** (0.2968)
**Controls**	Yes	Yes	Yes	Yes	Yes
**Id**	30	30	30	30	30
**Obs**	480	480	480	480	480
**R-sq**	0.8905	0.8853	0.8916	0.9292	0.8889

### Extended discussion: targeting mechanism of financial inclusion supporting farmers’ income increase

Financial inclusion, as an important mechanism to support farmers’ income increase, how to achieve accurate targeting is the basic premise to improve the efficiency of financial resource allocation and enhance the effect of supporting farmers’ income increase. In the view of financial sociologists, credit right is the concentrated expression of financial right, and credit exclusion is a serious injury to the basic rights of the poor (Morduch, 2000) [27]. Therefore, financial inclusion has been mainly committed to solving the credit exclusion faced by vulnerable groups such as farmers for a long time, making financial services from abstract rights provisions into a realistic means of exercising rights. However, as the financial demand side, poor farmers are often subject to the constraints of material capital, human capital, labor skills, economic behavior, etc. even in the case of sufficient financial supply, their own characteristics will restrict their effective use of financial resources and services to expand productive activities, and it is difficult to directly benefit from the increase of financial availability (Zheng and Zhu, 2019) [28]. In recent years, studies have also found that the financial inclusion empowerment development model has only a limited role in improving income distribution and reducing poverty and increasing income (Li and Han, 2017) [29]. The development mode of financial inclusion should turn to aim at the industrial development in rural areas, promote the local economic growth by supporting the industrial development, provide more employment opportunities for farmers, broaden their income sources, and solve the problem of unsustainable increase of farmers’ income from the source.

In view of China’s basic national conditions and the basic situation of rural areas, in practice, how should financial inclusion promote the development of agricultural industry in order to play a more effective role in driving farmers’ income? Division of labor economy is the driving force of increasing returns to scale and economic growth. The main reason why the increase of agricultural labor productivity can not keep up with the increase of manufacturing labor productivity is that it is difficult for agriculture to implement a complete division of labor system (Smith,1776) [30]. The lack of deepening agricultural division of labor is an important reason for the low efficiency of agricultural production. The driving force of the transformation of China’s agricultural growth mode is to improve the level of division of labor among agriculture, rural areas and farmers (Lou et al., 2012) [31]. With the progress of agricultural technology and the development of artificial intelligence, agricultural production has a certain material basis to promote the improvement of production efficiency through the division of labor and scale expansion. Financial inclusion should become an important means to promote the deepening of agricultural division of labor, and bring farmers into the agricultural industrial chain through the mode of division of labor, transaction and social cooperation, so as to improve the opportunities for farmers to share economic achievements. The development pole theory was proposed by Perroux (1955) [32]. The core idea of the theory is that the economic development of a region takes the lead in individual regions or leading departments, and then the development of these leading departments or regional economies will drive the entire regional economic development. The theory of development pole has certain applicability for financial inclusion to promote the development of agricultural industry. China’s rural resources are limited and the development between regions is extremely unbalanced. We can concentrate the limited resources in the region to support the development of the local leading industries with regional comparative advantages, and promote the continuous development of the leading industries through the industrialized operation and operation, and finally drive the economic development of rural areas through the agglomeration and diffusion effect of the development pole. In this process, financial inclusion can become a promoter to activate and organically combine various factors of production, and provide systematic credit support for the development of rural leading industries and the construction of related infrastructure.

To sum up, we believe that the targeting mechanism of financial inclusion to support farmers’ income increase should be based on the satisfaction of farmers’ credit rights, pay more attention to solving the problems of low efficiency of financial resource allocation and commercial sustainability, strive to stimulate the endogenous power and self-development ability of farmers and other vulnerable groups, and promote the development of agricultural industrialization based on division of labor and cooperation.

## Conclusions and policy recommendations

Using the data of 30 provinces in China from 2006 to 2022, we empirically test the impact of financial inclusion on farmers’ income growth, and explore the transmission mechanism of financial inclusion promoting farmers’ income growth. We find that: (1) Financial inclusion can significantly promote the growth of farmers’ income. Every unit of financial inclusion level is increased, farmers’ income will increase by 0.1337 units. (2) The regression results show that financial inclusion can significantly promote the growth of farmers’ wage income, family business income, farmers’ property income and transfer income; Compared with low-income farmers, financial inclusion has stronger promotion to high-income farmers; Financial inclusion promotes farmers’ income more strongly in areas with better infrastructure and higher information level. (3)Financial inclusion can increase farmers’ income by promoting agricultural industrialization, and the intermediary effect of agricultural industrialization can explain 1.7432% of the total effect of financial inclusion on increasing farmers’ income, which indicates that agricultural industrialization is the transmission mechanism of financial inclusion on increasing farmers’ income. (4) The interaction effect of financial inclusion and agricultural industrialization is not significant. This shows that the improvement of financial inclusion has not enhanced the marginal effect of agricultural industrialization on farmers’ income, and there are some structural problems in China’s financial inclusion. Further analysis shows that financial inclusion has played an effective role in supporting agricultural intensification, but the support for agricultural integration needs to be improved.

Our empirical results have the following implications for policy-makers and financial service providers. First of all, financial institutions should consider the necessity and feasibility of further increasing business outlets and self-service equipment in rural areas, pay special attention to the availability and use effect of financial products and services for farmers, and design differentiated financial products and services according to the characteristics of different farmers to better meet their diversified financial needs. Secondly, financial institutions should intensify the research and development of financial innovation technologies and their application in the agricultural sector, promote the upgrading of inclusive financial service quality, encourage rural areas to shift from self-sufficient small-scale peasant production mode to agricultural industrialization production mode based on division of labor and cooperation (Hu et al., 2021) [33], guide farmers and other vulnerable groups into the production track of agricultural industrialization, and provide systematic credit support for the development of agricultural industrialization in rural areas. Meanwhile, the government should focus on fostering local leading enterprises and characteristic industrialized organizations, continuously increase the number of farmers’ professional cooperatives, gradually release the demand for agricultural finance, and force the innovation and transformation of financial inclusion. Thirdly, rural infrastructure can not only reduce the production costs of various links in agricultural production, but also help promote the flow of all production factors, including capital, talent, and technology, between different regions or industrial sectors in rural areas. Financial institutions should strengthen deep cooperation with relevant agricultural enterprises and government departments, grasp the current situation and shortcomings of rural infrastructure investment, and focus on investing credit funds in infrastructure projects such as water conservancy facilities, rural roads, and circulation facilities that play a key role in the development of agricultural industrialization, providing a good software and hardware environment for the development of agricultural industrialization. Finally, financial institutions and government departments should strengthen financial education for farmers (Sayinzoga et al., 2016) [34], increase publicity and training of financial knowledge, enhance rural residents’ risk prevention awareness and financial legal awareness, improve rural residents’ understanding of financial inclusion products and services, cultivate rural residents’ ability to acquire financial knowledge, guide them to establish correct financial concepts, improve farmers’ ability to use credit funds for investment and production operations, and reduce their self exclusion of financial products and services.

However, there are some limitations that might be addressed by analysis in the future. First, there is room to expand the sample size. Due to the limited availability of data, we only select data at the provincial level. We hope to dig deeper into data at municipal and county level on this topic for further research. Secondly, there are relatively few scholars studying the agricultural industrialization from the perspective of rural areas, and thus there is a lack of sufficient reference basis to measure agricultural industrialization more accurately. Future research could build a more perfect agricultural industrialization measurement system. Finally, the indicators involved in financial inclusion index constructed in this paper are based on previous research results, China’s actual situation and the availability of relevant data, and the financial inclusion index system is not perfect enough. Therefore, choosing more reasonable and comprehensive indicators to improve the financial inclusion index system is also an important aspect of future research.

## Supporting information

S1 Data Raw data. (XLS)

S2 Data Basic data. (XLS)
